# Age-Related Alterations in Brain Perfusion, Venous Oxygenation, and Oxygen Metabolic Rate of Mice: A 17-Month Longitudinal MRI Study

**DOI:** 10.3389/fneur.2020.00559

**Published:** 2020-06-12

**Authors:** Zhiliang Wei, Lin Chen, Xirui Hou, Peter C. M. van Zijl, Jiadi Xu, Hanzhang Lu

**Affiliations:** ^1^Russell H. Morgan Department of Radiology and Radiological Science, Johns Hopkins University School of Medicine, Baltimore, MA, United States; ^2^F. M. Kirby Research Center for Functional Brain Imaging, Kennedy Krieger Research Institute, Baltimore, MA, United States; ^3^Department of Biomedical Engineering, Johns Hopkins University School of Medicine, Baltimore, MA, United States

**Keywords:** aging, cerebral blood flow, cerebral metabolic rate of oxygen, longitudinal, C57BL/6

## Abstract

**Background:** Characterization of physiological parameters of the aging brain, such as perfusion and brain metabolism, is important for understanding brain function and diseases. Aging studies on human brain have mostly been based on the cross-sectional design, while the few longitudinal studies used relatively short follow-up time compared to the lifespan.

**Objectives:** To determine the longitudinal time courses of cerebral physiological parameters across the adult lifespan in mice.

**Methods:** The present work examined longitudinal changes in cerebral blood flow (CBF), cerebral venous oxygenation (Y_v_), and cerebral metabolic rate of oxygen (CMRO_2_) using MRI in healthy C57BL/6 mice from 3 to 20 months of age. Each mouse received 16 imaging sessions at an ~1-month interval.

**Results:** Significant increases with age were observed in CBF (*p* = 0.017) and CMRO_2_ (*p* < 0.001). Meanwhile, Y_v_ revealed a significant decrease (*p* = 0.002) with a non-linear pattern (*p* = 0.013). The rate of change was 0.87, 2.26, and −0.24% per month for CBF, CMRO2, and Y_v_, respectively. On the other hand, systemic parameters such as heart rate did not show a significant age dependence (*p* = 0.47). No white-matter-hyperintensities (WMH) were observed on the T_2_-weighted image at any age of the mice.

**Conclusion:** With age, the mouse brain revealed an increase in oxygen consumption. This observation is consistent with previous findings in humans using a cross-sectional design and suggests a degradation of the brain's energy production or utilization machinery. Cerebral perfusion remains relatively intact in aged mice, at least until 20 months of age, consistent with the absence of WMH in mice.

## Introduction

Characterization of neurobiological changes in the aging brain is important in understanding brain function, neurodegeneration, and possibly dementia (1–3). While brain anatomical and structural measures have been studied extensively and generally reveal degradations with age (4–8), functional and physiological changes are more complex and have yielded more intriguing observations (9–12). For example, many studies on human brain aging have shown that fMRI signals in response to tasks are paradoxically greater in old than in young participants (13–16). Furthermore, when measuring basal brain oxygen metabolic rate as a surrogate marker of neural activity at rest, several studies have suggested that the brain of an older individual consumes more oxygen (per unit volume tissue) compared to younger individuals (10, 17). These findings have led to several interesting hypotheses such as a compensatory increase in neural activity in older individuals (10, 18), a diminishment of specificity in brain activation (19), or a reduced computational efficiency in neural circuits (20).

However, a shared limitation of these reports lies in their cross-sectional experimental design without the tracking of same subjects over time. There are also concerns related to potential sampling bias, in that the older participants may represent a group of “supernormals” who can stay healthy and participate in research even at an advanced age. While a few other studies have been conducted in a longitudinal fashion, the follow-up duration was relatively short compared to the lifespan (21–23), which may hamper their ability to estimate changes over longer periods of time.

In preclinical studies, mouse has been a popular species due to its genetic pliability to develop mutant strains and availability for surgery to build disease models (24). In particular, its proximity of genome and physiology to human and short lifespan (1~3 years) make it an excellent candidate for aging investigations (25, 26). Therefore, a longitudinal study on age-related changes in brain physiological parameters of mice will shed new light on neurobiological alterations in aging.

Brain relies on aerobic metabolism to provide energy for neuronal activities (27), thus the oxygen consumption constitutes an interesting parameter to understand the neurobiology of the aging brain. In this study, we examined longitudinal changes in physiological parameters of oxygen supply (i.e., cerebral blood flow, CBF), venous oxygenation (Y_v_), and oxygen consumption (i.e., cerebral metabolic rate of oxygen, CMRO_2_). These physiological parameters were evaluated with quantitative MRI techniques in C57BL/6 mice from 3 to 20 months old at an approximately 1-month interval. To allow the mice to survive the large number of imaging sessions, no MRI contrast agents were used and all measures were based on non-contrast-agent MRI sequences. In addition to the physiological measures, T_2_-weighted images were also collected for the assessment of potential white-matter hyperintensities, which are thought to be associated with insufficient blood supply (28).

## Materials and Methods

### General

The experimental protocols involved in this study were approved by the Johns Hopkins Medical Institution Animal Care and Use Committee, and conducted in accordance with the National Institutes of Health guidelines for the care and use of laboratory animals. Five C57BL/6 mice (female, Charles River Laboratories) were scanned longitudinally from 3 to 20 months old at an interval of 1 month. Two of the monthly scans were not completed due to the unavailability of scanner caused by technical problems. Thus, 16 time points (3~9, 11~14, and 16~20 months old) were collected.

One mouse was euthanized after the 16-month-old time point for ethical reasons due to the animal's suffering from a rectal prolapse. Behavioral test using a Y-maze was performed in the remaining mice at ~20 months of age (i.e., the last time point) to investigate the spatial memory as a representative for cognition status at this advanced age. A spontaneous-alternating-performance score in percent was obtained from the test (29).

### MRI

All MRI experiments were performed on an 11.7T Bruker Biospec system (Bruker, Ettlingen, Germany) equipped with a horizontal bore and actively shielded pulsed field gradients (maximum intensity of 0.74 T/m). Images were acquired using a 72-mm quadrature volume resonator as a transmitter and a four-element (2 × 2) phased-array coil as a receiver. The B_0_ field over the mouse brain was homogenized by a global shimming (up to 2nd order) based on a pre-acquired subject-specific field map.

Respiration rate was monitored during the experiment to ensure the survival of the mouse. Anesthesia was administered under following regimen: 1.5% vaporized isoflurane was used for 15 min as the induction followed by a continuous 1.0% isoflurane for maintenance until the end of experiments; during the experiment, the maintenance dosage would be increased slightly to ~1.2% in case that a mouse breathed at a rate >150 breaths per minute. At the 10th minute under 1.5% isoflurane inhalation, the mouse was immobilized with a bite bar and ear pins, and placed onto a water-heated animal bed with temperature control before entering the magnet.

Each MRI session consisted of MRI measurements detailed below.

### Cerebral Venous Oxygenation (Y_v_)

Y_v_ was assessed non-invasively from the confluence of sagittal sinuses with a T_2_-relaxation-under-spin-tagging (TRUST) technique, which was originally developed on human scanners and recently optimized for animal MRI systems (30–32). In order to visualize the confluence of sagittal sinuses, an axial time-of-flight (TOF) sequence was first performed with the following parameters: TR/TE = 20/2.6 ms, field of view (FOV) = 16 × 16 mm, matrix size = 256 × 256, 35 axial slices, slice thickness = 0.5 mm, and scan duration = 2.2 min. The TRUST scan was then positioned based on the TOF images (31), and was repeated three times to improve precision. The TRUST sequence used the following parameters: TR/TE = 3500/6.5 ms, FOV = 16 × 16 mm, matrix size = 128 × 128, slice thickness = 0.5 mm, EPI factor = 16, inversion-slab thickness = 2.5 mm, post-labeling delay = 1000 ms, eTE = 0.25, 20, 40 ms, echo spacing of eTE = 5.0 ms, and scan duration = 2.8 min.

**Figure 1:**
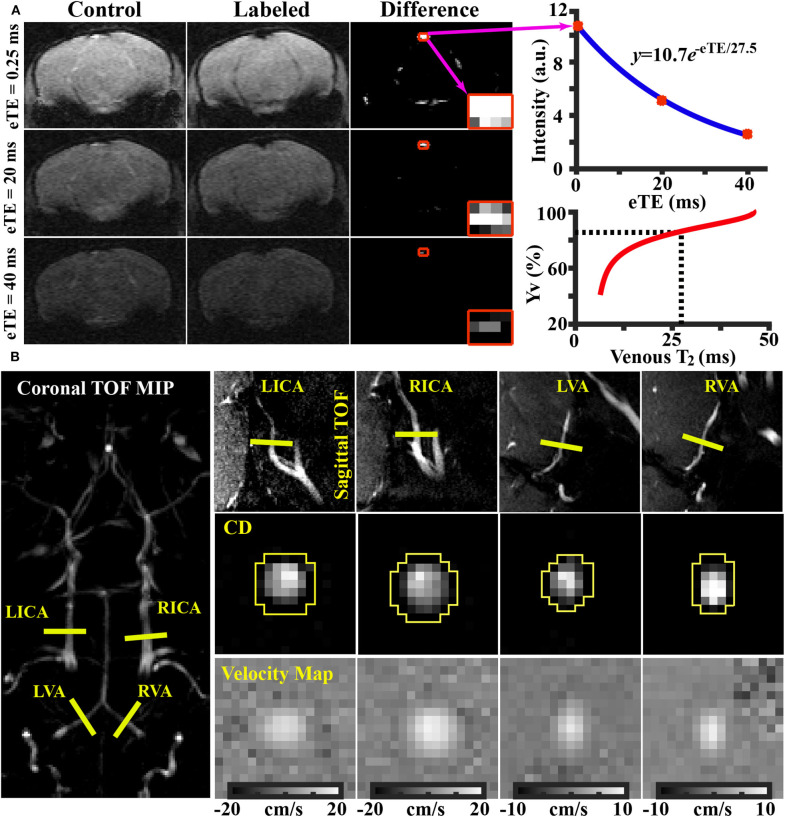
Representative results of **(A)** TRUST and **(B)** PC MRI. **(A)** Left panel: control, labeled, and difference images from TRUST MRI obtained at different effective TE (eTE) values. The sinus confluence is shown in red squares. Right upper panel: Blood signal in the sinus confluence as a function of eTE. Blue curve indicates fitting results. Right lower panel: Y_v_-T_2_ calibration plot used to convert blood T_2_ to oxygenation (33). **(B)** Left panel: imaging slice locations of PC MRI overlaid on a TOF image. Right upper panel: PC MRI imaging locations overlaid on respective sagittal TOF image. Right middle panel: complex difference (CD) images of the four arteries. Right lower panel: velocity maps of the four arteries.

Data processing of TRUST MRI was conducted with a custom-written graphic-user-interface (GUI) tool built on MATLAB (MathWorks, Natick, MA) and followed procedures detailed previously (30, 31). Briefly, for each TRUST dataset, subtraction between the control and labeled images was performed to obtain difference images (Figure 1A). A region of interest (ROI) was manually drawn on the difference image to encompass the sinus confluence. Four voxels within the ROI with the largest difference signals were selected for spatial averaging. Then, venous blood signal intensities at three different eTEs were fitted into a monoexponential function to obtain T_2_ (Figure 1A). Finally, T_2_ was converted into Y_v_ using a T_2_-Y_v_ calibration plot (Figure 1A) reported by Li et al. (33).

### Cerebral Blood Flow (CBF)

Global CBF was evaluated with phase-contrast (PC) MRI covering four major feeding arteries (Figure 1B), i.e., left internal carotid artery (LICA), right internal carotid artery (RICA), left vertebral artery (LVA), and right vertebral artery (RVA), in separate scans to collect corresponding through-plane velocity maps (Figure 1B) (34, 35). Prior to the PC scans, we first performed a coronal TOF angiogram (7 slices, slice thickness = 0.5 mm, no inter-slice gap, TR/TE = 45/2.6 ms, scan duration = 2 min) to visualize the feeding arteries. Then, a sagittal TOF (single slice, tilted to contain the target artery identified from coronal TOF images, thickness = 0.5 mm, TR/TE = 60/2.5 ms, scan duration = 0.4 min) was applied to visualize the in-plane trajectory of the target artery. PC MRI was then positioned using both TOFs and performed using following parameters: TR/TE = 60/3.2 ms, FOV = 15 × 15 mm, matrix size = 300 × 300, slice thickness = 0.5 mm, number of average = 4, dummy scan = 8, receiver bandwidth = 100 kHz, flip angle = 25°, partial Fourier acquisition factor = 0.7, and scan duration = 2.4 min.

Processing of PC dataset was performed with custom-written MATLAB scripts (MathWorks, Natick, MA). The artery of interest was first manually delineated on the complex-difference image (Figure 1B), which shows an excellent contrast between vessel and surrounding tissue. The mask was then applied to the phase velocity map and the integration of arterial voxels yields blood flow through that targeted artery in ml/min. Summation of blood-flow values across the four major feeding arteries yields total blood flow to the brain. To further account for the brain-size differences and to obtain unit-mass CBF values, the total blood flow was divided by the brain weight, which was calculated as the product of brain volume and density [1.04 g/ml (36)]. The global CBF value is written in the unit of milliliters per 100 g brain tissue per minute (ml/100 g/min). Inter-rater reproducibility of PC MRI processing has previously been assessed and reported an interrater correlation of >95% (34).

### Cerebral Metabolic Rate of Oxygen (CMRO_2_)

CMRO_2_ was computed from Y_v_ and CBF using the Fick principle (37–39), i.e., CMRO_2_ = *C*_*a*_ · (*Y*_*a*_ − *Y*_*v*_) · *CBF*, where C_a_ denotes the molar concentration of oxygen in a unit volume of blood and was assumed to be 882.1 μmol O_2_/100 ml blood based on previous literature (40, 41). Y_a_ represents arterial oxygen saturation fraction. Y_a_ is generally close to unity, and is assumed to be 0.99 in this study (42). CMRO_2_ is written in the unit of μmol oxygen per 100 g brain tissue per min (μmol O_2_/100 g/min).

### Additional Anatomical MRI Sequence

A T_2_-weighted fast spin-echo MRI protocol was utilized to collect anatomical MRI images. The imaging parameters were: TR/TE = 4000/26 ms, FOV = 15 × 15 mm, matrix size = 128 × 128, slice thickness = 0.5 mm (without inter-slice gap), echo spacing = 5 ms (8 spin echoes per scan), 35 axial slices, and scan duration = 1.1 min (43, 44).

In humans, microvascular insults to the brain are often assessed by white matter hyperintensities (WMH) on T_2_-weighted images (45–47). We therefore visually inspected the T_2_-weighted images to examine the potential presence of hyperintensities. Z.W. (>5 years of experience) and J. X. (>10 years of experience) performed independent image assessments and reached a consensus.

Additionally, the T2-weighted images were analyzed manually by delineating the brain boundary on a slice-by-slice basis (by Z.W.) while referencing to a mouse brain atlas (https://atlas.brain-map.org/). Voxels inside the masks were summed to yield the total brain volume in mm^3^. The total brain volume was used in the estimation of unit-mass CBF as described above.

### Heart Rate Measurement

It is known that heart rate is inversely related to the dosage of isoflurane anesthesia in rodents (48). We therefore utilized a MRI sequence to perform in-scanner measurement of heart rate during each session. The sequence acquired the center *k*-space repeatedly at an interval of 8.0 ms, thereby yielding a time course of MR signal intensity, the period of which is the R-R interval (49). Two measurements were performed in each session and the values were averaged. This allowed us to examine whether there is an age-dependent change in anesthesia level and how this may affect the interpretation of the physiological data.

### Statistical Analyses

All statistical analyses were performed with SPSS v23 (IBM Corporation, Armonk, NY). A linear mixed-effect model was utilized to analyze longitudinal measurements (i.e., Y_v_, CBF, CMRO_2_, and heart rate), in which age was a fixed effect and individual mouse was a random effect. An age^2^ term was also tested in the model to determine any non-linear effect of age. A *p*-value < 0.05 was considered significant.

## Results

Figure 1A shows representative data from TRUST MRI, which consisted of control, labeled, and difference images acquired at different eTE values. Figure 1B illustrates representative PC images. Both complex difference (CD) and velocity map images are shown. ROIs (yellow polygons in Figure 1B) delineating the target vessels are also displayed.

**Figure 2:**
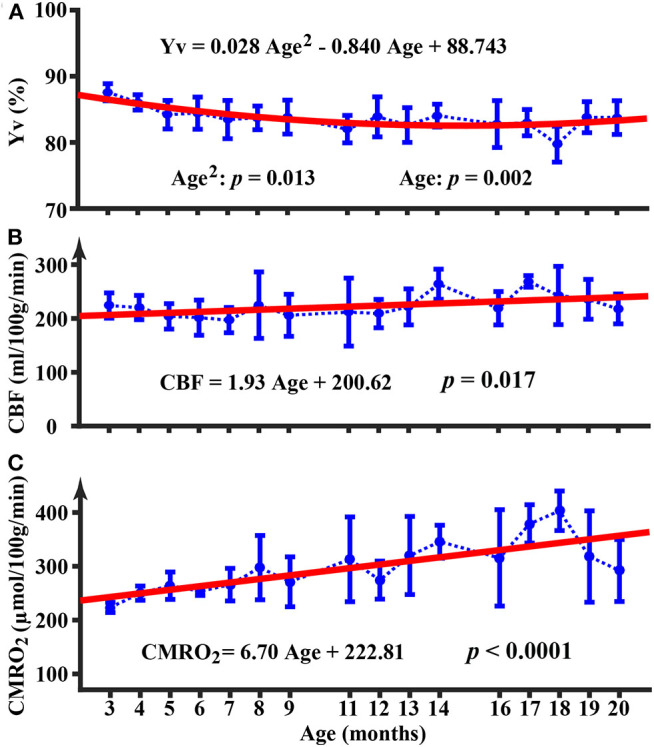
Longitudinal time courses of physiological parameters of Y_v_ **(A)**, CBF **(B)**, and CMRO_2_ **(C)**. Error bar denotes the standard deviation across mice (*N* = 5). Red line indicates the fitting curve from a mixed-effect model. Equation shows the fixed term estimated from the mixed-effect model.

The longitudinal time course for Y_v_ is shown in Figure 2A. There was an age-related decrease in Y_v_ (*p* = 0.002) from 3 to 20 months of age. Furthermore, the quadratic term of age was also significant (*p* = 0.013), suggesting that the age-dependence of Y_v_ was non-linear (${\text{Y}}_{v}=0.028{\text{Age}}^{2}-0.840\text{Age}+88.743$). Figure 2B displays the longitudinal time courses of CBF, which exhibited an increase with age (*p* = 0.017) (*CBF* = 1.93Age + 200.62 ml/100g/min). There was not a quadratic effect of age on CBF measure (*p* = 0.64). Longitudinal time course of CMRO_2_ is shown in Figure 2C and exhibited an increase with age (*p* < 0.0001) (*CMRO*_2_ = 6.70Age + 222.81 μmol/100g/min). The Age^2^ term was not significant for CMRO_2_ (*p* = 0.12). At 19 and 20 months of age, there appeared to be a decrease in CMRO_2_; but these values were not significantly different from CMRO_2_ at 18 months of age (*p* = 0.11 and 0.09, respectively).

**Figure 3:**
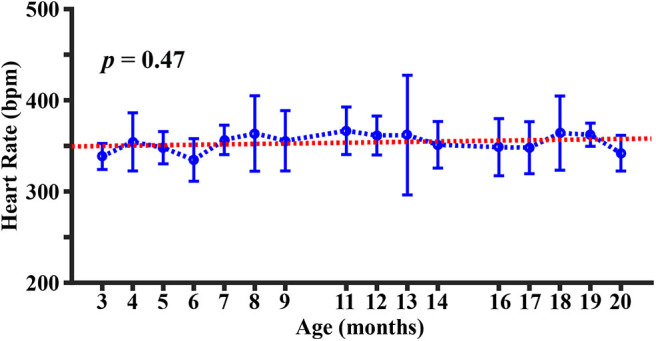
Longitudinal time course of heart rate. Error bar denotes the standard deviation across mice (*N* = 5). Red line indicates the fitting curve from a mixed-effect model.

Figure 3 shows the longitudinal time course of heart rate. It can be seen that the heart rate is within the range of 300~400 beats per minute (bpm), which is consistent with those reported in the literature under similar anesthetic conditions (50). There was no significant change in heart rate with age (*p* = 0.47).

The Y-maze test revealed an average spontaneous-alternating-performance score of 62 ± 20%. These scores are within the normal range of wild-type mice (29, 51), indicating that the utilized mice did not exhibit obvious cognitive decline throughout the study period.

**Figure 4:**
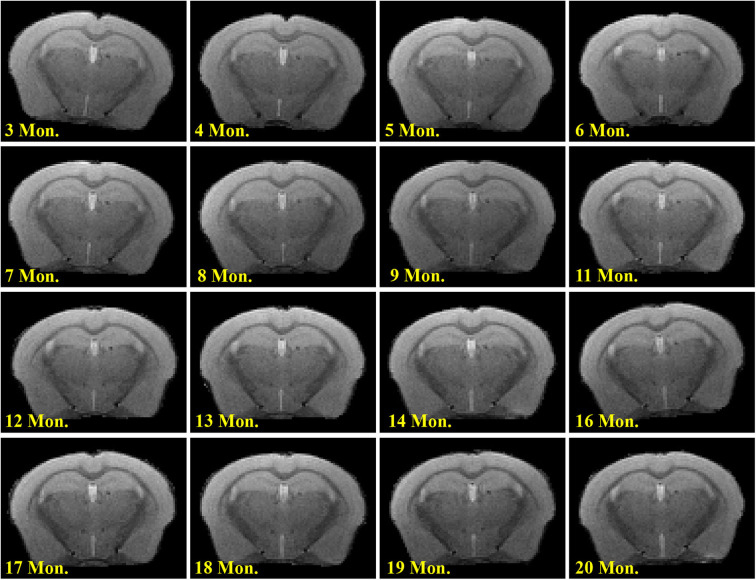
Longitudinal T_2_-weighted images in a representative mouse.

Visual inspection of T_2_-weighted images revealed no WMH in the brain, even at 20 months of age (Figure 4).

## Discussion

To the best of our knowledge, the present work is the first longitudinal study to characterize brain energy homeostasis across the adult lifespan of mice. Our findings suggested that brain perfusion and oxygen metabolic rate both increased within the age range of 3 to 20 months in C57BL/6 mice, consistent with the notion of flow-metabolism coupling (52). On the other hand, systemic parameters such as heart rate did not show a significant change with age. Results from the present study also provide normative data on these important brain physiological parameters of C57BL/6 mice, which is the most widely-used mouse strain for developing disease models (53, 54).

Our data suggested that CMRO_2_, an index of the brain's energy budget and a surrogate marker of aggregated brain cell activity, is in the range of 250–350 μmol/100 g/min in mice. These values are in good agreement with reports in the literature using ^17^O magnetic resonance spectroscopy (55, 56) or Doppler/spectroscopic OCT methods (40), and are higher than human CMRO_2_ values of 120–200 μmol/min/100 g, showing consistency with reports that mouse brain consumes more energy (per unit volume tissue) compared to humans (57, 58). Our longitudinal data also confirmed previous cross-sectional observations in humans that CMRO_2_ does in fact increase with age (10, 17). In terms of the reason for age-related increase in oxygen metabolism, it could be a compensatory response to reduced efficiency in neural computation and/or cellular machinery of oxidative metabolism and energy production chain. It has been reported that 24 month-old rats reveal ≥30% (59) synapse losses in comparison with 3-month-old young controls. Therefore, it is plausible that escalated neural computation is needed in the presence of synaptic loss.

Quantification of CBF in mice is not trivial. The present study revealed, for the first time, that unit-volume CBF increases with age in mice. Compared to the literature, our CBF values of 200–250 ml/100 g/min in mice are in good agreement with those obtained with arterial-spin-labeling (ASL) MRI, which showed values of ~200 ml/min/100 g for mice at 3 months of age (60, 61). However, previous ASL studies in mice have generally failed to observe an age effect on CBF. For example, Maier et al. performed a longitudinal ASL study (1.5, 2.5, 5, 7, 13, and 18 months old) in mice and found that CBF was constant across age (62). Similarly, Hirschler et al. reported in a cross-sectional ASL study that no CBF difference was observed between mice 6 and 26 months of age (63). Note that the ASL technique relies on the spin tagging principle to generate perfusion information after pair-wise subtraction (64). A limitation of ASL MRI is that the SNR of this signal is relatively low, thus it is typically used in a lower resolution setting in humans. In the mouse brain, spatial resolution has to be increased for delineation of structures, which results in a further decrease in SNR in mouse ASL data. In addition, ASL MRI also suffers from multiple confounding factors, e.g., bolus arrival time, labelling efficiency, and vessel contributions, in terms of its quantification. On the other hand, PC MRI utilizes the principle that flowing spins can accumulate a phase proportional to its velocity through the use of a pair of bipolar gradients (65). Two separate scans with inverted gradient polarities can be performed to cancel phases due to spurious field inhomogeneities and thereby allows the calculation of a flow map. The quantification of blood flow in PC is more straightforward with fewer confounding factors. Moreover, the coefficient of variation (CoV) of PC (5.3%) (34) was found to be smaller than that in ASL (~10%) (66), suggesting that PC is more reproducible. Therefore, the present study used the PC MRI technique to quantify global CBF, and the sequence has been extensively characterized previously for quantitative flow measurement (34, 67). CBF measurement using PC MRI presents a new approach for hemodynamic assessment in mice.

In humans, it has been suggested that CBF decreases with age (10, 68, 69). This decrease is thought to be attributed to increased arterial stiffness, thickening of basement membrane, stenosis or narrowing of vessel lumen, and development of arteriolosclerosis (70, 71). The present observation of an age increase in CBF of mice is apparently different from the human findings. One possible explanation is species differences. Humans have a much longer lifespan and thus vascular degradation may be substantial in older individuals. In contrast, it is possible that cerebral vessels are still in good health in aged mice. This is consistent with the histology study where cerebral capillary density was not significantly different between 24-month-old and 7-month-old C57BL/6 mice (72), and the notion that aged mice rarely have strokes (73) or WMH as shown in the present study. Therefore, when brain metabolic rate increases with age, mouse brain is able to garner more blood flow to meet its demand, following the flow-metabolism coupling principle (52). It is also interesting to compare CBF time-pattern in mice to that in humans for the same absolute time period. Previous studies that investigated CBF changes in neonates and young children have shown that, the first few months of humans, CBF exhibited an increase from ~20 ml/100 g/min to ~70 ml/100 g/min from 30 to 120 gestational weeks (i.e., 7 to 28 months) (74). Thus, it is plausible that CBF can increase in the first two years of life, and this occurs in both humans and mice.

One potential confounding factor in animal studies like ours is the anesthetic effect on physiological parameters. In this study, the anesthetic regimen used was the same throughout the study period. To further confirm that the age changes observed in this work were not attributed to variations in anesthetic level, we measured heart rate as an index of anesthetic depth. As shown in Figure 3, the heart rate did not reveal a dependence on age, indicating that the anesthetic level was not a contributor to the physiological alternations observed in the present study. This is consistent with literature that resting heart rate does not change with age (75). Isoflurane is known to induce an increase in CBF and a decrease in CMRO_2_ (76). Therefore, absolute values of metabolic parameters during awake state may be different from those reported here. Based on the relationship among CBF, CMRO2, and OEF, Yv measured under isoflurane is expected to be greater than that under awake state. Additionally, it should be pointed out that different anesthetic agents may induce different physiological alterations. For example, dexmedetomidine has been reported to be associated with decreases in both CBF and CMRO_2_ (77). Therefore, absolute values of physiological parameters under different anesthesia are not directly comparable; however, the age pattern is expected to be valid when a consistent anesthetic scheme is used.

Human studies have reported that Y_a_ is minimally affected by age with a 1~2% decrease across the lifespan (10). On the other hand, noninvasive measurement of Y_a_ in mouse is not trivial, and the measurement error could easily exceed the systematic error in Y_a_. Regarding C_a_, it is related to the hemoglobin level, which is not significantly different between young and older age according to the literature (78). Therefore, the assumptions on Y_a_ and C_a_ are not expected to alter the major conclusions in the current study.

Findings in current study should be interpreted in the context of several limitations. First, brain perfusion and oxygen consumption were measured in a global manner without spatial information. Regional maps can further enhance our understanding of spatial distributions of oxygen metabolism across the brain. Second, arterial oxygenation was based on an assumed value (99%) instead of subject-specific measurements, e.g., with pulse oximeter. The CMRO_2_ estimation is thus based upon such assumptions. However, we should point out that our study used a longitudinal design thus inter-subject variations in Y_a_ should not affect our conclusion. Finally, this study has only used female C57BL/6 mice. Thus potential sex effects on our findings require further studies. The number of mice used in this study was relatively small. However, we would like to point out that, when it comes to study power, one should also consider the number of data points from each mouse. In the present study, each mouse underwent 16 sessions (one with 12 sessions). Thus the total number of experimental sessions was 76, which is considered large compared to many studies in the literature. As a result, the statistical power of our findings was satisfactory, with a *p*-value of 0.002 for Y_v_ and <0.0001 for CMRO_2_. In addition, there are limitations of using animal models to study human conditions. In this case, the aging process in humans is expected to be different from that in mice in many aspects (79–81). Aged mice often do not develop neurodegeneration and have low prevalence of cardiovascular disease, possibly due to species differences in physiology, disparity in maximal lifespan, diet, and environmental factors. Thus caution should be used when generalizing these observations to other mouse strains.

## Conclusions

We conducted a longitudinal assessment of brain physiological parameters in C57BL/6 mice, and observed an age-related increase in brain perfusion and oxygen consumption within 3 to 20 months of age. While the age increases in oxygen metabolic parameters are consistent with findings in humans and suggest a compensatory hypermetabolism of brain tissue, the continuous increase in perfusion suggests that cerebrovascular function remains relatively intact in aged C57BL/6 mice and is consistent with an absence of WMH, in contrast to human elderly individuals.

## Data Availability Statement

The datasets generated for this study are available on request to the corresponding author.

## Ethics Statement

The animal study was reviewed and approved by Johns Hopkins Medical Institution Animal Care and Use Committee.

## Author Contributions

ZW and HL designed the study. ZW, LC, and JX performed the experiments and collected the data. XH helped with the statistical analyses. PZ and HL helped with the data interpretation. ZW and HL wrote the manuscript with editing from all other authors.

## Conflict of Interest

The authors declare that the research was conducted in the absence of any commercial or financial relationships that could be construed as a potential conflict of interest.
